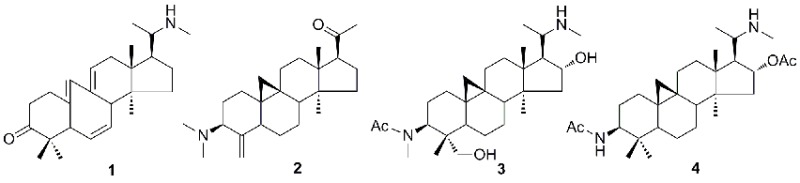# Triterpenoid alkaloid derivatives from *Buxus rugulosa*

**DOI:** 10.1007/s13659-011-0010-z

**Published:** 2011-09-21

**Authors:** Yu-Xin Yan, Lin Zhou, Yun Sun, Jian-Chao Chen, Jia Su, Yan Li, Ming-Hua Qiu

**Affiliations:** 1 State Key Laboratory of Phytochemistry and Plant Resources in West China, Kunming Institute of Botany, Chinese Academy of Sciences, Kunming, 650201 China; 2 Graduate University of Chinese Academy of Sciences, Beijing, 100039 China

**Keywords:** *Buxus rugulosa*, triterpenoid alkaloid derivatives, buxruguline, cytotoxicity

## Abstract

Four new triterpenoid alkaloid derivatives, buxrugulines A–D (**1–4**), together with four known ones (**5–8**), were isolated from the leaves and stems of *Buxus rugulosa*. The structures of compounds **1–4** were elucidated by NMR and MS spectroscopic analysis. All compounds were assayed for their cytotoxicities against HL-60, SMMC-7721, A549, MCF-7, and SW480 cells lines.

**Introduction**

*Buxus rugulosa*, belonging to the *Buxus* genus of the family Buxaceae, is a dwarf shrub growing in the rocky mountains in the northwest district of Yunnan Province. In previous phytochemical investigations of the genus *Buxus*, more than 220 triterpenoid alkaloid derivatives have been isolated[1,2]. This type of alkaloid showed interesting pharmacological activities such as anti-myocardial ischemia[3,4], antibacterial activities[5,6], and inhibition of cholinesterases[7–9]. In our previous studies from *Buxus* plants, new alkaloids with diverse structures and promising cytotoxic activities have been reported[10,11]. As part of this study, we have examined the stems and leaves of *B. rugulosa*, and consequently isolated four new triterpenoid alkaloid derivatives, buxrugulines A–D (**1**–**4**), along with four known ones, $N_{20}$-acetoxy-cyclovirobuxin D (**5**)[12], (+)-16$\alpha$-acetoxybuxabenzamidienine (**6**)[13,14], moenjodaramine (**7**)[9,15,16], irehine (**8**)[17]. Herein we report the isolation and structural elucidation of the new compounds, as well as cytotoxic activities of the isolates from *B. rugulosa*.

**Results and Discussion**

A crude alkaloid fraction of *B. rugulosa* yielded eight triterpenoid alkaloid derivatives by repeated silica gel, amino silica gel, C-18 and Sephadex LH-20 chromatography.

Buxruguline A (**1**) was obtained as white powder. Its molecular formula, $C_{25}H_{37}NO$, was established on the basis of HRESIMS analysis ($[M + H]^+$, *m/z* 368.2944). The $^1H$ NMR (Table 1) spectrum featured one N-methyl singlet at $\delta_H$ 2.47, four singlets at $\delta_H$ 0.80, 1.05, 1.17, and 1.26, corresponded to four tertiary methyl groups, and one doublet at $\delta_H$ 0.76 (6.5, H-21). The $^{13}C$ NMR spectrum exhibited 25 carbon signals containing six quaternary carbons (one carbonyl carbon at $\delta_C$ 206.8 and two olefinic carbons at 146.1 and 146.3), eight methines (four olefinic carbons at $\delta_C$ 127.6, 127.7, 128.4, and

**Figure 1.** Structures of **1**–**8**.

*To whom correspondence should be addressed. E-mail: mhchiu@mail.kib.ac.cn

129.7), five methylenes, and six methyl groups. Comparison of the spectroscopic data of **1** and cyclobuxotriene[13] revealed similarities except for the absence of a methyl on the nitrogen at C-20 and the presence of a double bond at C-6/7 in **1**. This was supported by the HMBC correlations of H-5 ($\delta_H$ 2.73) with C-6 ($\delta$c 128.4) and of H-8 ($\delta_H$ 1.91) with C-7 ($\delta$c 127.6), C-9 ($\delta$c 146.1) (Figure 2). Therefore, **1** was elucidated as shown, and named buxruguline A.

**Figure 2.** Key HMBC correlations for compound **1**.

Buxruguline B (**2**) was obtained as colorless needles. The HRESIMS exhibited a quasi-molecular ion peak at *m*/*z* 370.3105 ([M + H]$^+$, calc. 370.3109), indicating the molecular formula $C_{25}H_{39}NO$. The $^1$H NMR spectrum featured three singlets for the three tertiary methyl groups at $\delta_H$ 0.85, 0.90, and 1.08, and the characteristic cyclopropyl methylene protons appeared as two doublets at $\delta_H$ − 0.04 and 0.50 (4.1). The $^1$H and $^{13}$C NMR spectrum of **2** displayed the presence of a terminal methylidene [$\delta_H$ 4.61, 4.85 (each, 1H, s); $\delta_H$ 153.3 (C), 101.5 ($CH_2$)]. All the data indicated that compound **2** was similar to buxpiine[7], and the distinct difference between them was that a oxygenated methine ($\delta_C \approx$ 72) of C-16 in buxpiine was replaced by a methylene ($\delta_C$ 34.2) in **2**. This deduction was supported by HMBC correlations from H-21 ($\delta_H$ 1.08) to C-20 ($\delta_C$ 212.9), C-16 ($\delta_C$ 34.2) and from H-17 ($\delta_H$ 2.66) to C-20, C-16 and C-13 ($\delta_C$ 42.7). H-5 is invariably $\alpha$-oriented in this type alkaloid[18,19], the ROESY correlation of H-3 ($\delta_H$ 2.85) with H-5 ($\delta_H$ 2.12) indicating an $\alpha$-orientation of H-3 and $\beta$-orientation of the amino functionality. So, the structure of **2** was elucidated as shown in Figure 1.

Buxruguline C (**3**) had the molecular formula $C_{28}H_{48}N_2O_3$, as determined by HRESIMS analysis ([M + H]$^+$, *m*/*z* 461.3749). The $^1$H NMR ($CDCl_3$) spectrum of **3** showed the presence of three methyl singlets at $\delta_H$ 1.08, 0.60, and 0.92 for the H-18, H-31, and H-32, a doublet methyl at $\delta_H$ 1.01 (6.0, 21-$CH_3$), two N-methyl singlets at $\delta_H$ 2.45 and 2.88, together with characteristic protons due to one hydroxymethylene ($\delta_H$ 2.93 and 3.25), and one oxygenated methine ($\delta_H$ 4.27). These spectral data were quite similar to those of dihydrocyclobaleabuxine[20], except for the resonance of acetyl group [$\delta_H$ 1.92; $\delta_C$ 172.2 (C) and 22.3 ($CH_3$)] attributable to the nitrogen at C-3. The relative configuration of **3** was elucidated by the ROESY experiment and comparision with other naturally occurring triterpenoid alkaloid possessing $\beta$-configuration of the amino group at C-3, H-5$\alpha$, and H-20$\beta$[18,19]. The ROESY correlations of H-5 ($\delta_H$ 1.84), and H-3 ($\delta_H$ 2.98) with H-30 ($\delta_H$ 2.93 and 3.25), and of H-20 ($\delta_H$ 2.97) with H-16 ($\delta_H$ 4.27) indicated that H-30 was in $\alpha$-orientation, while H-16 was in $\beta$-orientation, respectively. Thus, the structure of buxruguline C was established as **3**.

The moleculr formula of buxruguline D (**4**) was assigned as $C_{29}H_{48}N_2O_3$ on the basis of the NMR data (Table 1) and HRESIMS. Comparison of the spectroscopic data of **4** and **3** revealed similarities cycloartane-type triterpenoid skeleton. The notable difference was that a OH functionality at C-16 in **3** was replaced by acetoxy group in **4**, which confirmed by the downfielded H-16 ($\delta_H$ 4.11) proton signal and the HMBC correlation from H-16 to the O-acetyl carbonyl carbon at $\delta_C$ 169.3 (C). Moreover **4** has one less hydroxyl function at C-30 and one less methyl group on the nitrogen at C-3 than **3**. Consequently, compound **4** was elucidated as shown and has been accorded the trivial name buxruguline D.

Biologically, all compounds were tested for their cytotoxicity against the HL-60, SMMC-7721, A549, MCF-7, and SW480 cells lines (Table 2). Compounds **6**, **7** and **8** showed the better cytotoxic potential against A-549, and SW480 cell lines. Compounds **1–4** were noncytotoxic, with $IC_{50}$ values > 40 $\mu$mol for all tested cell lines.

## Experimental Section

**General Experimental Procedures.** Melting points were determined on a YU-HUA X-4 melting point apparatus. Optical rotations were obtained with a Horiba SEAP-300 polarimeter. Infrared spectra were recorded on a Shimadzu IR-450 instrument by using KBr pellets. NMR spectra were measured on a Bruker AV-400 and DRX-500 instrument (Bruker, Zűrich, Switzerland) with TMS as internal standard. HR-ESIMS data were recorded on a VG Auto Spec-3000 spectrometer. Silica gel (200–300 mesh, Qingdao Marine Chemical, Inc), amino silica gel (75−100 $\mu$m, Fuji Silysia Chemical LTD, Japan), C-18 (20–45 $\mu$m, Fuji Silysia Chemical, LTD, Japan), and Sephadex LH-20 (Pharmacia) were used for column chromatography.

**Plant Material.** *Buxus rugulosa* were collected at Lijiang (Yunnan), China, in February 2008. The sample was identified by Prof. Xi-Wen Li of the Kunming Institute of Botany, and a voucher specimen (KIB 20080210) has been deposited at the State Key Laboratory of Phytochemistry and Plant Resources in West China, Kunming Institute of Botany, Chinese Academy of Sciences.

**Extraction and Isolation.** The materials of *B. rugulosa* (75.0 kg) were extracted with 90% MeOH under reflux, the combined extracts were partitioned between EtOAc and 0.001 mol/L HCl (pH $\approx$ 3.0). The aqueous layer was alkalinized to pH 10.0 with 2 mol/L NaOH followed by exhaustively extraction with $CHCl_3$. The $CHCl_3$-soluble fraction (180 g) was chromatographed on a silica gel column, eluted with $CHCl_3$-MeOH (1:0-0:1), to give four fractions (FA–FD). FB (45 g) was chromatographed on silica gel using petroleum ether (PE)-EtOAc (8:1) as solvent and repeated Sephadex LH-20 eluted with MeOH to yield **7** (19 mg). After column chromatography on C-18 gel column chromatography by aqueous MeOH (60%-90%), amino silica gel column with PE-EtOAc (10:1) and $CHCl_3$-MeOH (50:1), further separated by Sephadex LH-20 eluted with MeOH, FC (14 g) to afford **1** (11 mg), **4** (4 mg), **5** (6 mg), **7**(28 mg), **8** (42 mg). FD (12 g) was chromatographed on silica gel using $CHCl_3$-MeOH (10:1, 5:1) as gradient, and was further repeatedly separated on amino silica gel column chromatography, eluted with $CHCl_3$-MeOH (20:1, 10:1), to give **2** (6 mg), **3** (12 mg), **6** (76 mg).

**Buxruguline A (1):** white powder; mp 188–190°C; $[\alpha]_D^{24}$ + 15.6 (*c* 1.04, $CHCl_3$); UV (MeOH) $\lambda_{max}$ (log ε) 208 (3.78), 244 (2.21) nm; IR (KBr) $\nu_{max}$: 1734 $cm^{-1}$; $^1H$, $^{13}C$ NMR data see Table 1; EIMS *m/z*: 367, HRESIMS *m/z*: 368.2944 $[M + H]^+$ (calcd for $C_{25}H_{38}NO$ $[M + H]^+$, 368.2953).

**Buxruguline B (2):** colorless needle; mp 223–224 ˚C; $[\alpha]_D^{24}$ + 18.4 (*c* 1.21, $CHCl_3$); UV (MeOH) $\lambda_{max}$ (log ε) 205 (3.59) nm; IR (KBr) $\nu_{max}$: 1735 $cm^{-1}$; $^1H$, $^{13}C$ NMR data see Table 1; ESIMS *m/z*: 370 $[M + H]^+$; HRESIMS *m/z* 370.3105 (calcd for $C_{25}H_{40}NO$ $[M + H]^+$, 370.3109).

**Table 1. $^1H$ and $^{13}C$ NMR Spectral Data of Buxrugulines A–D (1–4).**

| | **1**[a] | | **2**[b] | | **3**[c] | | **4**[a] | |
|---|---|---|---|---|---|---|---|---|
| position | $\delta_C$, type | $\delta_H$ | $\delta_C$, type | $\delta_H$ | $\delta_C$, type | $\delta_H$ | $\delta_C$, type | $\delta_H$ |
| 1a | 29.7, $CH_2$ | 2.04, overlap | 31.0, $CH_2$ | 1.82, overlap | 32.4, $CH_2$ | 1.56, overlap | 31.6, $CH_2$ | 1.65, overlap |
| 1b | | 1.90, overlap | | 1.46, overlap | | 1.17, overlap | | 1.47, overlap |
| 2a | 46.6, $CH_2$ | 2.34, m | 25.7, $CH_2$ | 1.73, overlap | 27.0, $CH_2$ | 1.55, overlap | 25.6, $CH_2$ | 1.33, overlap |
| 2b | | 1.84, m | | 1.65, m | | | | 1.08, overlap |
| 3 | 206.8, C | | 63.6, CH | 2.85, m | 51.3, CH | 2.98, overlap | 65.5, CH | 3.75, m |
| 4 | 44.3, C | | 153.3, C | | 44.0, C | | 42.2, C | |
| 5 | 41.1, CH | 2.73, d (3.5) | 41.9, CH | 2.12, m | 40.2, CH | 1.84, s | 50.5, CH | 2.16, s |
| 6a | 128.4, CH | 5.62, m | 21.2, $CH_2$ | 1.80, overlap | 20.5, $CH_2$ | 1.62, overlap | 20.9, $CH_2$ | 1.50, overlap |
| 6b | | | | 1.18, overlap | | | | 0.76, overlap |
| 7a | 127.6, CH | 5.42, m | 26.2, $CH_2$ | 1.58, overlap | 25.4, $CH_2$ | 1.22, overlap | 28.4, $CH_2$ | 1.75, m |
| 7b | | | | 1.14, overlap | | 1.12, overlap | | 1.49, overlap |
| 8 | 41.5, CH | 1.91, overlap | 42.4, CH | 2.00, m | 47.5, CH | 1.35, overlap | 47.3, CH | 1.44, overlap |
| 9 | 146.1, C | | 23.3, C | | 19.0, C | | 19.6, C | |
| 10 | 146.3, C | | 32.0, C | | 25.5, C | | 25.8, C | |
| 11a | 127.7, CH | 5.69, m | 21.4, $CH_2$ | 1.50, overlap | 25.8, $CH_2$ | 2.04, m | 26.1, $CH_2$ | 1.33, overlap |
| 11b | | | | 1.16, overlap | | 1.04, overlap | | 1.08, overlap |
| 12a | 30.2, $CH_2$ | 1.60, overlap | 33.3, $CH_2$ | 1.72, overlap | 31.7, $CH_2$ | 1.62, overlap | 32.2, $CH_2$ | 1.22, overlap |
| 12b | | 1.50, m | | 1.55, overlap | | 1.44, overlap | | |
| 13 | 39.5, C | | 42.7, C | | 45.6, C | | 44.9, C | |
| 14 | 41.5, C | | 46.0, C | | 47.7, C | | 48.0, C | |
| 15a | 24.8, $CH_2$ | 2.04, overlap | 22.4, $CH_2$ | 1.52, overlap | 45.5, $CH_2$ | 1.82, overlap | 45.0, $CH_2$ | 1.38, overlap |
| 15b | | 1.58, overlap | | 1.18, overlap | | 1.34, overlap | | |
| 16a | 26.0, $CH_2$ | 1.32, overlap | 34.2, $CH_2$ | 2.12, m | 75.8, CH | 4.27, m | 78.5, CH | 4.11, m |
| 16b | | | | 1.17, overlap | | | | |
| 17 | 47.5, CH | 1.88, overlap | 46.8, CH | 2.66, m | 56.1, CH | 1.93, m | 57.0, CH | 1.88, m |
| 18 | 19.7, $CH_3$ | 0.80, s | 18.3, $CH_3$ | 0.85, s | 18.9, $CH_3$ | 1.08, s | 19.0, $CH_3$ | 0.97, s |
| 19 | 129.7, CH | 6.51, s | 22.0, $CH_2$ | 0.50, d (4.1) | 30.0, $CH_2$ | 0.51, d (4.1) | 30.2, $CH_2$ | 0.53, d (4.1) |
| | | | | − 0.04, d (4.1) | | 0.32, d (4.1) | | 0.35, d (4.1) |
| 20 | 68.1, CH | 2.09, m | 212.9, C | | 64.1, CH | 2.97, overlap | 62.7, CH | 2.71, m |
| 21 | 15.3, $CH_3$ | 0.76, d (6.5) | 17.8, $CH_3$ | 1.08, s | 10.3, $CH_3$ | 1.01, d (6.0) | 9.8, $CH_3$ | 0.91, d (6.5) |
| 30a | 20.5, $CH_3$ | 1.26 (s) | 101.5, $CH_2$ | 4.85, s | 63.7, $CH_2$ | 3.25, m | 11.4, $CH_3$ | 0.76, s |
| 30b | | | | 4.61, s | | 2.93, m | | |
| 31 | 22.6, $CH_3$ | 1.17, s | | | 10.9, $CH_3$ | 0.60, s | 19.0, $CH_3$ | 0.97, s |
| 32 | 25.8, $CH_3$ | 1.05, s | 11.5, $CH_3$ | 0.90, s | 20.3, $CH_3$ | 0.92, s | 21.1, $CH_3$ | 1.15, s |
| 3-$NCH_3$ | | | 34.5, $CH_3$ | 2.50, s | 35.3, $CH_3$ | 2.45, s | | 2.30, s |
| 20-$NCH_3$ | 35.3, $CH_3$ | 2.47, s | | | 30.5, $CH_3$ | 2.88, s | | |
| N*C*O$CH_3$ | | | | | 172.2, C | | 171.4, C | |
| NCO*C*$H_3$ | | | | | 22.3, $CH_3$ | 1.92, s | 21.0, $CH_3$ | 2.09, s |
| O*C*O$CH_3$ | | | | | | | 169.3, C | |
| OCO*C*$H_3$ | | | | | | | 23.6, $CH_3$ | 1.96, s |

[a] $^1H$ and $^{13}C$ NMR spectra were acquired at 500 ($CDCl_3$) and 125 MHz ($CDCl_3$), respectively. [b] $^1H$ and $^{13}C$ NMR spectra were acquired at 400 ($CDCl_3$) and 100 MHz ($CDCl_3$), respectively. [c] $^1H$ and $^{13}C$ NMR spectra were acquired at 400 [$CDCl_3$:MeOD (1:1)] and 100 MHz [$CDCl_3$:MeOD (1:1)], respectively.

**Table 2. Cytotoxicity data of compounds 1–8 with $IC_{50}$ values ($\mu$M).**

| No. | HL-60 | SMMC-7721 | A-549 | MCF-7 | SW480 |
|---|---|---|---|---|---|
| **1** | > 40 | > 40 | > 40 | > 40 | > 40 |
| **2** | > 40 | > 40 | > 40 | > 40 | > 40 |
| **3** | > 40 | > 40 | > 40 | > 40 | > 40 |
| **4** | > 40 | > 40 | > 40 | > 40 | > 40 |
| **5** | 27.18 | > 40 | > 40 | 27.03 | > 40 |
| **6** | 15.23 | 28.99 | 19.39 | 14.39 | 14.69 |
| **7** | 17.32 | > 40 | 19.70 | > 40 | 14.25 |
| **8** | 21.35 | > 40 | 23.52 | > 40 | 17.18 |
| cisplatin (MW300) | 1.00 | 17.05 | 26.75 | 14.97 | 16.88 |

**Buxruguline C (3):** colorless needle; $[\alpha]_D^{24}$ + 8.8 (*c* 0.89, $CHCl_3$); UV (MeOH) $\lambda_{max}$ (log ε ) 205 (3.53) nm; IR (KBr) $\nu_{max}$: 1698 $cm^{-1}$; $^1H$, $^{13}C$ NMR data see Table 1; ESIMS *m/z* 461 $[M + H]^+$; HRESIMS *m/z* 461.3749 (calcd for $C_{28}H_{49}N_2O_3$ $[M + H]^+$, 461.3743).

**Buxruguline D (4):** white powder; $[\alpha]_D^{24}$ + 17.5 (*c* 0.72, $CHCl_3$); UV (MeOH) $\lambda_{max}$ (log ε ) 203 (3.67) nm; IR (KBr) $\nu_{max}$: 1696 $cm^{-1}$; $^1H$, $^{13}C$ NMR data see Table 1; EIMS *m/z* 472 $[M + H]^+$; HRESIMS *m/z* 473.7114 (calcd for $C_{29}H_{49}N_2O_3$ $[M + H]^+$, 473.7109).

**Cell Culture and Cytotoxicity Assay.** A panel of human tumor cell lines was used: promyelocytic leukemia HL-60, hepatocellular carcinoma SMMC-7721, alveolar basal epithelial carcinoma A549, breast adenocarcinoma MCF-7, and colon cancer SW480. The cells lines were obtained from the Shanghai cell bank of China. All the cells were cultured in RPMI-1640 or DMEM medium (Hyclone, USA), supplemented with 10% fetal bovine serum (Hyclone, USA) at 37°C in a humidified atmosphere with 5% $CO_2$.

Cell viability was assessed by conducting colorimetric measurements of the amount of insoluble formazan formed in the living cells with the MTT (MTT, sigma, USA) method described before[21], and using cisplatin (DDP, sigma, USA) as control. Cell growth inhibition curve was graphed and the $IC_{50}$ value of each compound was calculated by the Reed and Muench method[22].

## Acknowledgments

The project was financially supported by the Knowledge Innovation Program of the CAS (Grant No. KSCX2-YW-G-038, KSCX2-YW-R-194, KSCX2-EW-R-15, and KZCX2-XB2-15-03), as well as Foundation of State Key Laboratory of Phytochemistry and Plant Resources in West China (P2008-ZZ05 and P2010-ZZ14).

**Open Access** This article is distributed under the terms of the Creative Commons Attribution License which permits any use, distribution, and reproduction in any medium, provided the original author(s) and source are credited.
